# Synthesis and Application of Salicylhydrazone Probes with High Selectivity for Rapid Detection of Cu^2+^

**DOI:** 10.3390/molecules29092032

**Published:** 2024-04-28

**Authors:** Tianzhu Shi, Zhengfeng Xie, Xinliang Mo, Yulong Feng, Tao Peng, Fuyong Wu, Mei Yu, Jingjing Zhao, Li Zhang, Ju Guo

**Affiliations:** 1 Department of Brewing Engineering, Moutai Institute, Renhuai 564500, China; xinliangmo@163.com (X.M.); edifcztony@126.com (T.P.); wufuyong@mtxy.edu.cn (F.W.); yumei@mtxy.edu.cn (M.Y.); zhaojingjing0613@163.com (J.Z.); zhangli0026@126.com (L.Z.); guoju@mtxy.edu.cn (J.G.); 2 Oil & Gas Field Applied Chemistry Key Laboratory of Sichuan Province, College of Chemistry and Chemical Engineering, Southwest Petroleum University, Chengdu 610500, China; xiezhf@swpu.edu.cn

**Keywords:** Schiff bases, triphenylamine, acylhydrazone, fluorescent probe, copper ion

## Abstract

Using the aldehyde amine condensation procedure and the triphenylamine group as the skeleton structure, the new triphenylamine-aromatic aldehyde-succinylhydrazone probe molecule DHBYMH was created. A newly created acylhydrazone probe was structurally characterized by mass spectrometry (MS), NMR, and infrared spectroscopy (FTIR). Fluorescence and UV spectroscopy were used to examine DHBYMH’s sensing capabilities for metal ions. Notably, DHBYMH achieved a detection limit of 1.62 × 10^−7^ M by demonstrating exceptional selectivity and sensitivity towards Cu^2+^ ions in an optimum sample solvent system (DMSO/H_2_O, (*v*/*v* = 7/3); pH = 7.0; cysteine (Cys) concentration: 1 × 10^−4^ M). NMR titration, high-resolution mass spectrometry analysis, and DFT computation were used to clarify the response mechanism. Ultimately, predicated on DHBYMH’s reversible identification of Cu^2+^ ions in the presence of EDTA, a molecular logic gate was successfully designed.

## 1. Introduction

The copper ion (Cu^2+^) is an essential redox-active trace element for animals and plants, which mainly plays a role in nerve transmission, oxygen delivery, and redox reactions. However, when there is a large amount of Cu^2+^ in the environment, it will accumulate in the human body through the food chain. Excess Cu^2+^ accumulation in the body may lead to a variety of diseases [1,2,3]. The normal level of total copper in blood is 15.7–23.6 μM. The Chinese standard for drinking water specifies a limit of 15.7 μM for Cu^2+^, and the US Environmental Protection Agency (EPA) sets the limit for Cu^2+^ in drinking water at 20.0 μM [4]. Therefore, the convenient and rapid detection and treatment of copper ions are of great importance for environmental protection and human health [5,6].

Traditional methods for analyzing Cu^2+^ include spectrophotometry [7], atomic absorption spectroscopy [8], electrochemical analysis [9], inductively coupled plasma mass spectrometry [10], and isotope dilution [11]. However, these methods are limited by expensive equipment, complex operation procedures, professional personnel, and difficulty in real-time on-site monitoring. Therefore, it is necessary to find a convenient, low-cost, and real-time method for detecting ions. Compared to traditional Cu^2+^ analysis detection techniques, fluorescence probe detection has the advantages of good selectivity, low detection limits, low cost, simple operation, real-time monitoring, and fast response [12,13,14,15,16,17,18]. It has become one of the most popular Cu^2+^ analysis detection methods and a research hotspot in recent years [19,20,21,22].

The triphenylamine unit, with its propeller structure, excellent electron-donating ability, and easily modified molecular structure, can serve as an excellent emitting group for small molecular fluorescence probes and has become a research hotspot in recent years [19]. Based on our previous studies [23,24,25,26,27,28,29,30,31,32], trianiline is modified by introducing 4-bromo-2-hydroxybenzaldehyde through a Suzuki coupling reaction to increase its conjugation degree and electron-giving ability, improving its optical properties. It also undergoes condensation with salicylhydrazine to introduce Schiff base structures, enhancing its coordination ability with copper ions to achieve rapid detection and high selectivity. Ultimately, a novel Schiff base-type small molecular fluorescence probe molecule, N-((4-(diphenylamino)-3-hydroxy-[1,1-biphenyl]-4-yl)methylene)-2-hydroxybenzoylhydrazone (DHBYMH), which has a certain solvent effect and can efficiently and sensitively detect copper ions in different water samples, was designed and synthesized. Its reversible design allows for its use in molecular logic gates.

## 2. Results and Discussion

### 2.1. Solvent–Chromic Effect

**Figure 1:**
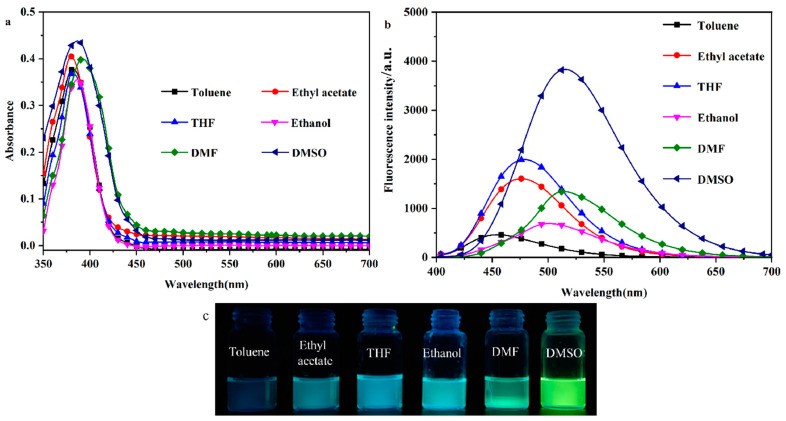
Absorption spectra (**a**) and emission spectra (**b**) of probe DHBYMH in toluene, ethyl acetate, tetrahydrofuran, ethanol DMF, and DMSO (composition of probe DHBYMH: 1.0 × 10^−5^ M; slit: 5/5). (**c**) Physical image of DHBYMH (1 × 10^−5^ M) in organic solvents of different polarity under a UV lamp (365 nm).

To investigate the solvent-induced color change effect of the probe molecule DHBYMH, the UV–Vis absorption spectra and fluorescence emission spectra of DHBYMH (1 × 10^−5^ M, 2 mL) were observed and recorded in different polar organic solvents (toluene, ethyl acetate, THF, ethanol, DMF, and DMSO). As shown in Figure 1a, there is no significant difference in the UV absorption spectra of the DHBYMH probe, with the maximum absorption wavelength around 385 nm. However, in the fluorescence emission spectra (Figure 1b), the DHBYMH probe exhibits significant differences in the six different polar organic solvents, which manifests as a marked redshift in the maximum emission wavelength with increasing solvent polarity. The maximum emission wavelengths are 454 nm in toluene, 476 nm in ethyl acetate, 479 nm in THF, 501 nm in ethanol, 514 nm in DMF, and 520 nm in DMSO, which are all higher than those of the DHBYMH probe in the same solvents. Across the whole range of solvent polarity, the experimental data do not obey the linear relationship predicted by the Lippert–Mataga equation well, as shown in Figure S2 and Table S1. It is worth noting that the quantum yield (QY) of the DPTYMH probe in DMSO is 25.06%, which is higher than that of other solvents. Figure 1c shows the actual images of DHBYMH (1 × 10^−5^ M) under UV light (365 nm) in the six organic solvents mentioned above. Table 1 lists the optical data from the DHBYMH probe in the six organic solvents, showing an increase in the Stokes shift with increasing solvent polarity. The main reason for the above phenomenon is that when the DHBYMH probe is excited by light, a π-π* transition occurs. During this transition, the excited state of the probe molecule usually decays or relaxes back to the ground state through non-radiative channels, causing the fluorescence quenching of π-conjugated molecules in the aggregated state and generating the aggregation-caused quenching (ACQ, Figure S1), resulting in an increase in the dipole moment of the excited state, which is higher than that of the ground state. This leads to an increase in the solvent–solute interaction force with increasing solvent polarity, resulting in a decrease in the excited state energy and a significant redshift in the spectrum. After mixing with Cu^2+^, the maximum absorption wavelength spectrum undergoes a significant redshift and the absorbance intensity increases slightly. The experimental phenomenon can be attributed to intramolecular charge transfer (ICT), which is induced by the π-bridge of the neighboring hydroxyphenyl ring in DHBYMH [33,34,35,36,37].

### 2.2. Cu^2+^ Response Behavior of DHBYMH

#### 2.2.1. Screening of Test Conditions

**Figure 2:**
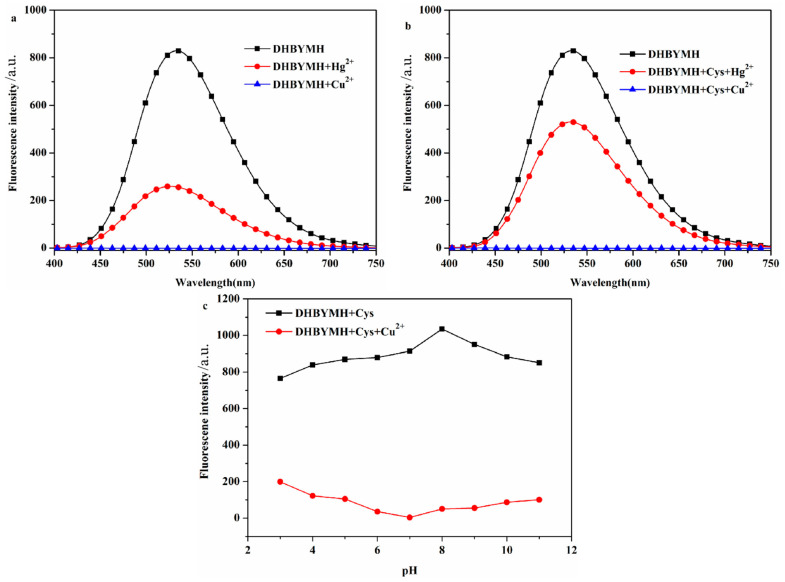
The probe DHBYMH (1 × 10^−5^ M, DMSO/H_2_O, *v*/*v* = 7/3) responded to Hg^2+^ and Cu^2+^ ions in the absence of Cys (**a**) and in the presence of Cys (1 × 10^−4^ M) (**b**). The fluorescence emission spectra of Cu^2+^ ion recognition by probe DHBYMH (1 × 10^−5^ M, DMSO/H_2_O, *v*/*v* = 7/3, pH = 7.0, Cys: 1 × 10^−4^ M) under different pH conditions (**c**).

In the optimized DMSO/H_2_O solvent system with a *v*/*v* ratio of 7/3, DHBYMH recognizes Cu^2+^ ions, and Hg^2+^ ions cause weak interference. However, in the presence of Cys, Hg^2+^ ions do not interfere with DHBYMH’s recognition of Cu^2+^ ions. Under the DMSO/H_2_O, *v*/*v* = 7/3, Cys: 1 × 10^−4^ M system, the ability of DHBYMH to recognize Cu^2+^ ions under different pH conditions was studied. It was found that in the pH range of 3–11, the probe DHBYMH exhibits fluorescence emission, and upon the addition of Cu^2+^ ions, a fluorescence quenching phenomenon occurs, with the maximum quenching degree at pH = 7 (Figure 2a–c). Therefore, the pH = 7 solvent system was selected for the exploration of the response time of the probe DHBYMH to Cu^2+^ ions. As shown in Figure S3, after stirring for 5 s, the probe DHBYMH reaches complete quenching, indicating an extremely fast response time of only 5 s. In summary, subsequent studies on DHBYMH’s recognition of Cu^2+^ ions were carried out under the conditions of DMSO/H_2_O, *v*/*v* = 7/3, pH = 7.0, Cys: 1 × 10^−4^ M, with a stirring time of 5 s.

#### 2.2.2. Selective Recognition of Cations 

**Figure 3:**
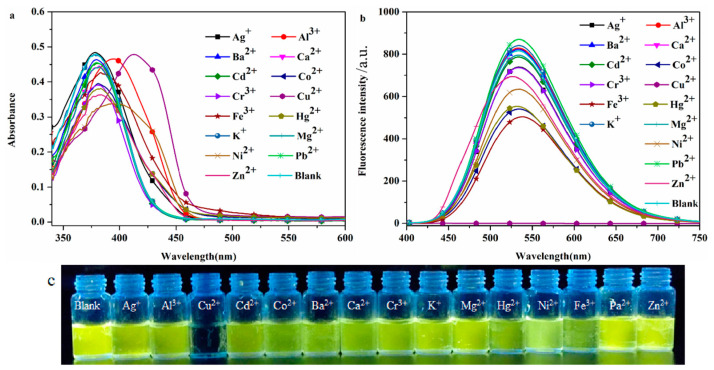
The UV absorption spectra (**a**) and fluorescence emission spectra (**b**) of the probe DHBYMH (1 × 10^−5^ M, DMSO/H_2_O, *v*/*v* = 7/3, pH = 7.0, Cys: 1 × 10^−4^ M) for selective recognition of common metal cations (1 × 10^−4^ M). (**c**) Physical image of DHBYMH (1 × 10^−5^ M, DMSO/H_2_O, *v*/*v* = 7/3, pH = 7.0, Cys: 1 × 10^−4^ M) for selective recognition of common metal cations (1 × 10^−4^ M) under UV lamp (365 nm) irradiation.

The high selectivity of the probe DHBYMH for the target ion is beneficial for practical applications. To investigate the selectivity of the probe DHBYMH for Cu^2+^ ions, absorption spectra and emission spectra were used to study the response of DHBYMH to common cations such as Ag^+^, Ba^2+^, Al^3+^, Ca^2+^, Co^2+^, Cd^2+^, Cu^2+^, Cr^3+^, Hg^2+^, Fe^3+^, Ni^2+^, K^+^, Mg^2+^, Pb^2+^, and Zn^2+^. Figure 3a shows that the maximum absorption peak of DHBYMH for Cu^2+^ ion recognition exhibited a significant redshift compared to other ions. In the emission spectrum (Figure 3b), DHBYMH only showed a significant response to Cu^2+^ ions, with complete quenching of fluorescence, while there was little response to other common metal ions. This phenomenon can also be observed by the naked eye under UV lamp (365 nm) illumination (Figure 3c). In summary, DHBYMH exhibits high selectivity for Cu^2+^ ions and has practical application value.

#### 2.2.3. Competitive Recognition of Cu^2+^ Ions 

**Figure 4:**
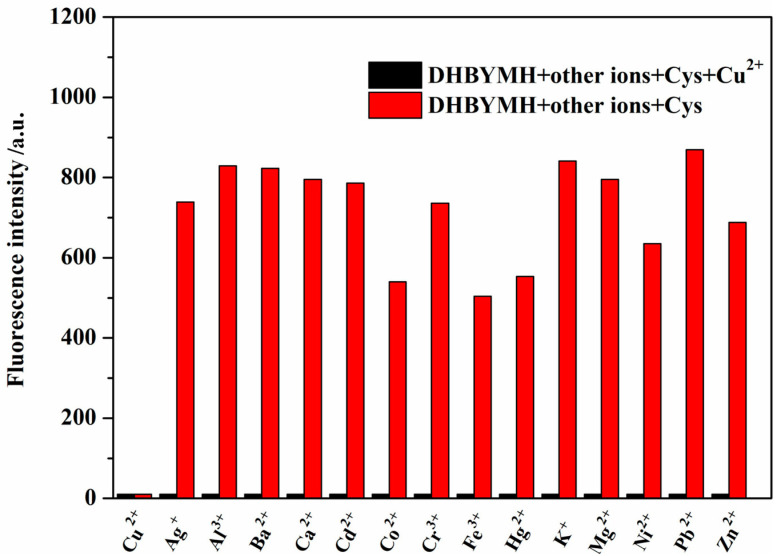
The detection of Cu^2+^ (1 × 10^−4^ M) by probe DHBYMH (1 × 10^−5^ M, DMSO/H_2_O, *v*/*v* = 7/3, pH = 7.0, Cys: 1 × 10^−4^ M) in the presence of other common metal ions was tested.

The better the interference resistance of the probe molecule, the stronger its competitive recognition ability for the target molecule and the higher its practical application value. Therefore, the recognition ability of DHBYMH for Cu^2+^ ions in the presence of common metal ions such as Ag^+^, Ba^2+^, Al^3+^, Ca^2+^, Co^2+^, Cd^2+^, Cu^2+^, Cr^3+^, Hg^2+^, Fe^3+^, Ni^2+^, K^+^, Mg^2+^, Pb^2+^, and Zn^2+^ was studied. As shown in Figure 4, DHBYMH still showed fluorescence emission in the presence of other common metal ions; however, a fluorescence quenching phenomenon occurred upon the addition of Cu^2+^ ions. Therefore, DHBYMH has good competitive recognition for Cu^2+^ ions, allowing for specific detection of Cu^2+^ ions in environmental systems.

#### 2.2.4. Determination of the Detection Limit of Probe DHBYMH for Cu^2+^ Ion Recognition 

**Figure 5:**
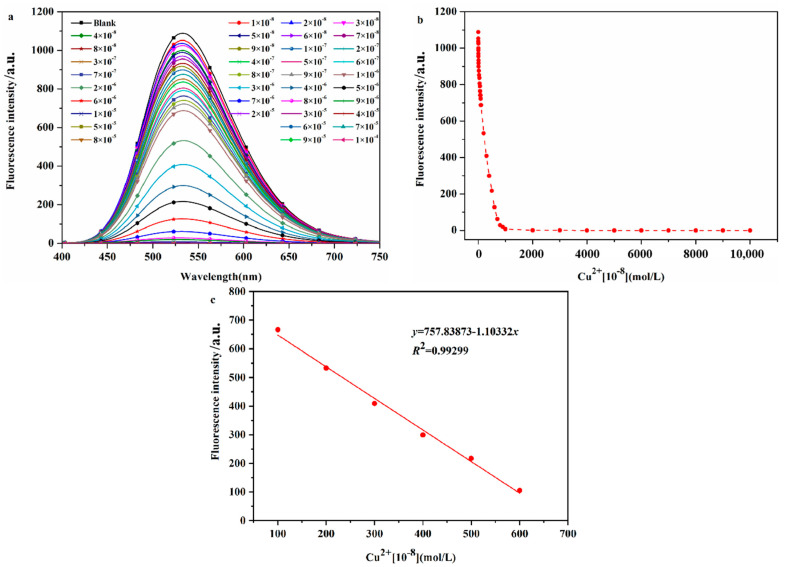
The fluorescence spectra (**a**) of different concentrations of Cu^2+^ ions (0~1 × 10^−4^ M) recognized by probe DHBYMH (1 × 10^−5^ M, DMSO/H_2_O, *v*/*v* = 7/3, pH = 7.0, Cys: 1 × 10^−4^ M), and the fluorescence dot diagram (**b**) of Cu^2+^ ions recognized by probe DHBYMH at 533 nm and at concentrations of 1 × 10^−6^~6 × 10^−6^ M with intervals defined by curve fitting (**c**).

The sensitivity of a probe molecule for the target ion is determined by its detection limit, and a high-sensitivity probe molecule has higher practical application value. Therefore, the detection limit of DHBYMH for Cu^2+^ ions was determined, as shown by the experimental results in Figure 5. As shown in Figure 5a, the fluorescence intensity of the probe DHBYMH (1 × 10^−5^ M) gradually decreased with increasing Cu^2+^ ion concentrations (0~1 × 10^−4^ M) until complete quenching occurred. Figure 5b shows the fluorescence dot plot of DHBYMH at an emission wavelength of 533 nm for different concentrations of Cu^2+^ ions. In the 1 × 10^−6^ to 6 × 10^−6^ M concentration range, the fluorescence intensity of DHBYMH showed a good linear relationship with the Cu^2+^ ion concentration, with a regression equation of y = 1037.019 − 1.01083x and a correlation coefficient of 0.99299 (Figure 5c). Based on the 3σ rule, the detection limit of DHBYMH for Cu^2+^ ions was calculated to be 1.62 × 10^−7^ M, which is lower than the limit of 20.0 μM for Cu^2+^ ions in drinking water set by the U.S. Environmental Protection Agency (EPA), indicating that this probe has good practical application value (compared with other studies, Table S2).

#### 2.2.5. The Mechanism of Cu^2+^ Ion Recognition 

**Figure 6:**
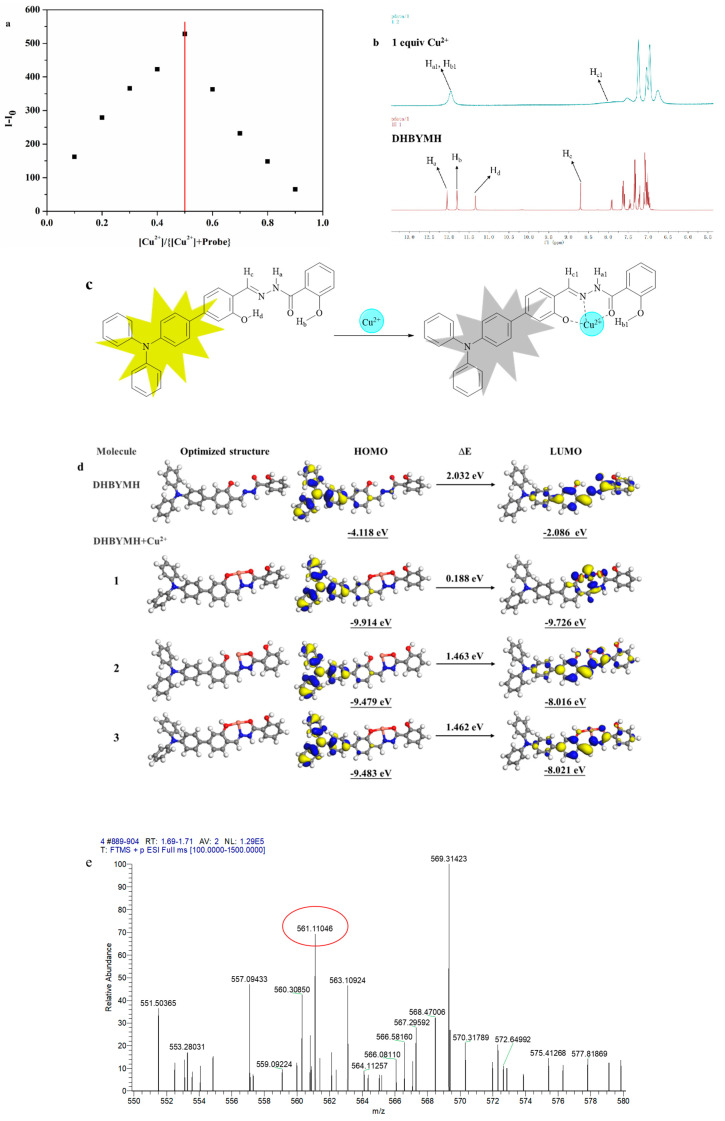
Job’s curve of Cu^2+^ ions recognized by probe DHBYMH (**a**). NMR titration diagram of probe DHBYMH and Cu^2+^ ions (**b**). Cu^2+^ ion recognition mechanism of probe DHBYMH (**c**). Optimal geometry of DHBYMH and DHBYMH+Cu^2+^ and electron cloud distribution of HOMO and LUMO levels (**d**). High-resolution mass spectrometry of DHBYMH+Cu^2+^ (**e**).

The mechanism of Cu^2+^ ion recognition by the probe DHBYMH was studied. The first step involved a Job’s plot experiment (Figure 6a), which determined the coordination ratio of the probe DHBYMH to Cu^2+^ ions to be 1:1. In the second step, a nuclear magnetic resonance (NMR) titration experiment was conducted, and the results showed that the protons H_a_, H_b_, H_c_, and H_d_ on the DHBYMH molecule experienced chemical shifts upon recognition by Cu^2+^ ions. H_a_ shifted from 12.05 ppm to 11.96 ppm, H_b_ shifted from 11.81 ppm to 11.96 ppm, the amino group’s H_a_ and hydroxyl group’s H_b_ merged into a single peak, H_c_ shifted from 8.70 ppm to 8.15 ppm, and the peak of H_d_ at 11.34 ppm disappeared. Based on this analysis, the Cu^2+^ ion recognition mechanism of DHBYMH involves Cu^2+^ coordinating with the N atom in the carbon–nitrogen double bond and the O atom in the carbonyl group to form a stable five-membered ring structure while also coordinating with the O atom in the hydroxyl group on the benzene ring connected to the triphenylamine group to form a stable six-membered ring structure (Figure 6b,c).

To validate the above conclusions, density functional theory (DFT) calculations were performed using the DMol 3 module in Materials Studio software 8.0. Figure 6d shows the electronic cloud distribution of the optimized geometric structures of DHBYMH and DHBYMH+Cu^2+^ in three coordination structures (with structure 1 being the coordination structure mentioned above). The HOMO orbital electronic cloud distribution of DHBYMH and the three coordination structures of DHBYMH+Cu^2+^ are essentially evenly distributed in the triphenylamine group and the adjacent benzene ring. However, in the LUMO orbital electronic cloud distribution, the electronic cloud distribution of DHBYMH+Cu^2+^ in structure 1 is more concentrated in the five-membered ring structure and six-membered ring structure formed by DHBYMH and Cu^2+^ coordination compared to DHBYMH alone and in structures 2 and 3 (Figure 6d). This is due to the paramagnetic properties of Cu^2+^ in structure 1, which cause changes in the electronic transitions of the probe molecule, leading to fluorescence quenching, which is consistent with the experimental phenomenon. In addition, the HOMO–LUMO band gap of DHBYMH+Cu^2+^ in the calculated structure 1 is 0.188 eV, which is lower than that of the calculated structures 2 and 3 (1.463 eV, 1.462 eV). The HOMO–LUMO band gap below DHBYMH (2.032 eV) indicates that the molecular structure of DHBYMH+Cu^2+^ in calculated structure 1 is the most stable among the four structures, that is, Cu^2+^ ions preferentially form calculated structure 1 when coordinating with DHBYMH. High-resolution mass spectrometry was used to further verify the coordination of DHBYMH with Cu^2+^ ions in structure 1. The molecular ion peak at m/z 561.11046 is consistent with the molecular weight of structure 1 [DHBYMH+Cu^2+^–H^+^], as mentioned in Figure 6e. This further validates the recognition mechanism shown in Figure 6c [38].

#### 2.2.6. Construction of Molecular Logic Gates

**Figure 7:**
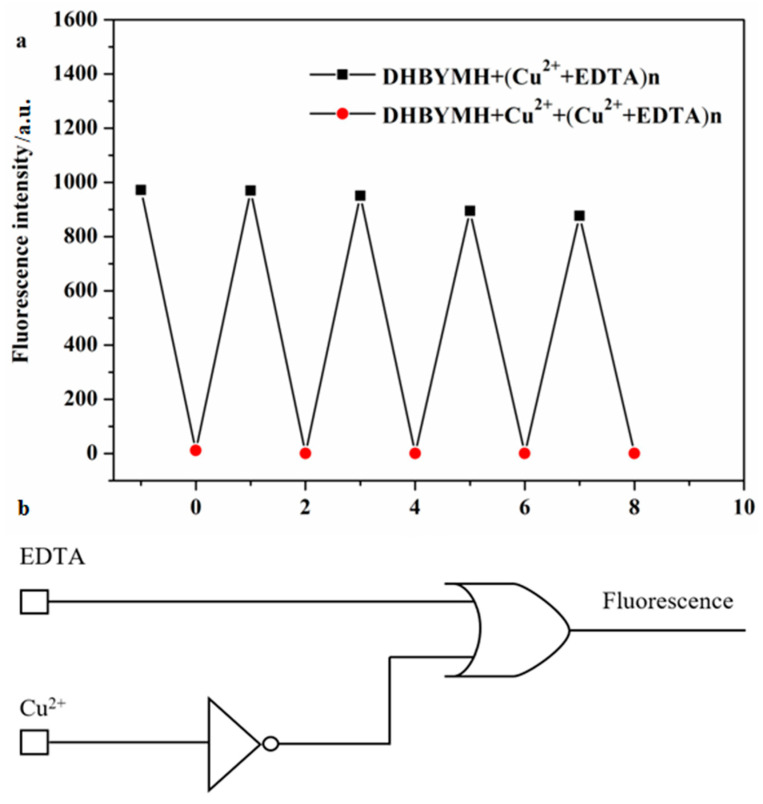
Reversible recognition of Cu^2+^ by probe DHBYMH (**a**). Molecular logic circuit diagram with Cu^2+^ and EDTA as chemical inputs and fluorescence intensity as chemical output (**b**).

The reversible recognition of target ions by fluorescent probes is beneficial because it enables more economical use of the probe molecules in practical applications. To study the reversible recognition performance of DHBYMH for Cu^2+^ ions, EDTA (ethylenediaminetetraacetic acid) can be used as a Cu^2+^ ion chelating agent to remove the Cu^2+^ ions that have already coordinated with DHBYMH, restoring the original fluorescence properties of DHBYMH. As shown in Figure 7a, the addition of EDTA to the DHBYMH+Cu^2+^ complex can restore the fluorescence of DHBYMH, and the addition of Cu^2+^ ions can quench the fluorescence again. After four cycles, DHBYMH still shows obvious fluorescence switching behavior. This indicates that DHBYMH has excellent reversibility and can be reused for detecting Cu^2+^ ions.

In recent years, designing molecular logic gates based on chemical substances has been an interesting topic in the study of electronic information technology and communication materials, which also provides new ideas for the practical application of probes [39,40,41]. Therefore, taking advantage of the reversibility of the probe DHBYMH, with Cu^2+^ ions and EDTA as chemical inputs and fluorescence intensity as the chemical output, a molecular logic gate was constructed. When inputting, Cu^2+^ ions and EDTA were added as 1, and their absence was 0; when outputting, the fluorescence emission was 1, and the fluorescence quenching was 0. According to the experimental data, the molecular logic gate truth table (Table 2) shows that when both Cu^2+^ ions and EDTA are absent (Entry 1) or only EDTA is present (Entry 2), DHBYMH shows fluorescence emission at 533 nm, outputting 1; when only Cu^2+^ ions are present (Entry 3), DHBYMH’s fluorescence is quenched, outputting 0; and when both Cu^2+^ ions and EDTA are present (Entry 4), DHBYMH shows fluorescence emission at 533 nm, outputting 1. Based on Table 2, a molecular logic circuit (Figure 7b) was designed.

## 3. Materials and Methods

### 3.1. Chemicals and Instruments

X-4 digital micromelting point determination instrument (Beijing TEC Instrument Co., Ltd., Beijing, China), Waters Q-TOF Premier time-of-flight mass spectrometer (WATERS Corporation, Milford, MA, USA), BRUKER 400 MHz nuclear magnetic resonance (Varian Inc., Palo Alto, CA, USA), DZF-6050 vacuum drying oven (Shanghai Hongdu Electronic Technology Co., Ltd., Shanghai, China), CP214 electronic analytical balance (Ohaus Instruments Co., Ltd., Shanghai, China), 78-1 magnetic heating stirrer (Shanghai Shuangjie Experimental Equipment Co., Ltd., Shanghai, China), YRE-2010 rotary evaporator (Gongyi City Yuhua Instrument Co., Ltd., Zhengzhou, China), TMS as an internal standard, CDCl_3_ or DMSO-*d*_6_ as solvents, UV-2450 type ultraviolet–visible spectrophotometer (Shimadzu Corporation, Kyoto, Japan), RF-5301PC fluorescence spectrophotometer (Shimadzu Corporation, Japan), and HORIBA Fluorolog-3 spectrophotometer (HORIBA Instruments Corporation, Irvine, CA, USA). All reagents used were analytical grade. Except for anhydrous ethanol and tetrahydrofuran, which were purified before use, other reagents were not further processed. The metal ions used, except for mercury acetate, were in the form of nitrate, and no further purification was performed before use.

### 3.2. Design and Synthesis

#### 3.2.1. Synthesis of 4-(Diphenylamino)-3-hydroxy-[1,1′-biphenyl]-4-formaldehyde (DHB) [42,43]

**Figure 8:**
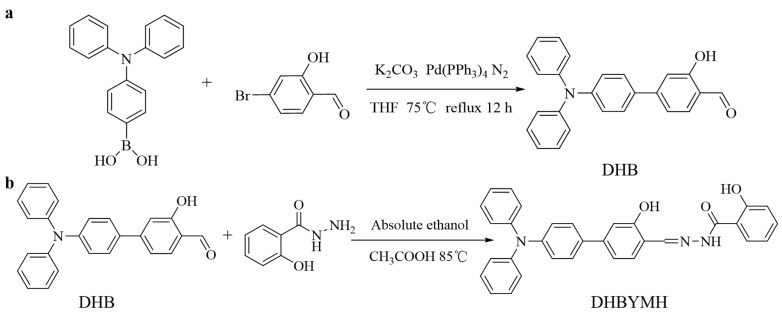
Synthetic routes of DHB (**a**) and DHBYMH (**b**).

Triphenylamine 4-borate (1 mmol), tetrakis (triphenylphosphine) palladium (Pd(PPh_3_)_4_, as catalyst), and 15.0 mL THF were added into a round-bottomed flask, and the reaction reflux was carried out for 0.5 h at 75 °C under the protection of N_2_. Then, 10.0 mL of THF solution dissolved in 4-bromo-2-hydroxybenzaldehyde (1.0 mmol) was added, and the reflux was continued at 75 °C under the protection of N_2_ for 12 h (Figure 8a). After cooling to room temperature, the mixture was poured into the liquid separation funnel and underwent demulsification with an appropriate amount of brine and then extraction with dichloromethane until the extraction liquid became colorless. The extracted organic phase was then dried with anhydrous sodium sulfate. Finally, the organic phase was separated and purified by column chromatography (elution ratio: ethyl acetate/petroleum ether, 1/30), and a solid greenish-yellow powder was obtained by rotary evaporation with a yield of 69%, which was DHB. M.p. 161–162 °C. FT-IR: 3434 cm^−1^; 3032 cm^−1^; 2834 cm^−1^; 1650 cm^−1^; 1591 cm^−1^; 1490 cm^−1^. ^1^H NMR (400 MHz, DMSO-*d*_6_) δ 10.84 (s, 1H), 10.22 (s, 1H), 7.73 (s, 1H), 7.62 (d, *J* = 8.8 Hz, 2H), 7.35 (dd, *J* = 8.4, 7.5 Hz, 4H), 7.23 (d, *J* = 10.5 Hz, 2H), 7.17–7.06 (m, 6H), 7.02 (d, *J* = 8.8 Hz, 2H). ^13^C NMR (101 MHz, DMSO-*d*_6_) δ 192.00, 161.57, 148.49, 132.19, 130.80, 130.17, 128.41, 125.19, 124.23, 122.73, 118.01, 114.41.

#### 3.2.2. The Synthesis of N-((4-(Diphenylamino)-3-hydroxy-[1,1-biphenyl]-4-yl)methylene)-2-hydroxybenzohydrazide (DHBYMH)

The DHB (0.5 mmol), salicylaldehyde (0.5 mmol), ethanol (8 mL, as solvent) and glacial acetic acid (0.5 mL, as catalyst) were added into a round-bottomed flask, and the mixture was refluxed at 85 °C for 4 h (Figure 8b). After cooling to room temperature, a solid precipitate was collected by filtration. The obtained solid precipitate was recrystallized using anhydrous ethanol, DHBYMH has been successfully synthesized as a yellow solid powder with a yield of 91%. M.p. 186–187 °C. FT-IR: 3343 cm^−1^; 3059 cm^−1^; 3032 cm^−1^; 1655 cm^−1^; 1628 cm^−1^; 1591cm^−1^; 1489 cm^−1^. ^1^H NMR (400 MHz, DMSO-*d*_6_) δ 12.05 (s, 1H), 11.80 (s, 1H), 11.34 (s, 1H), 8.70 (s, 1H), 7.93 (s, 1H), 7.73–7.54 (m, 3H), 7.46 (s, 1H), 7.38–7.31 (m, 4H), 7.23 (d, *J* = 10.7 Hz, 2H), 7.11–7.06 (m, 6H), 7.01 (dd, *J* = 16.5, 7.7 Hz, 4H). ^13^C NMR (101 MHz, DMSO-*d*_6_) δ 164.95, 159.54, 149.31, 147.38, 134.45, 133.09, 130.62, 130.11, 128.14, 124.92, 123.96, 123.22, 119.50, 117.79, 116.07, 114.00. HR-MS calculated for C_32_H_25_N_3_O_3_ [M + H]^+^ 500.19742 found 500.19653; HR-MS for [M + Na]^+^ 522.17936 found 522.17871.

### 3.3. DFT Calculations

All DFT calculations were performed using the DMol 3 module in Materials Studio software 8.0 [12,40]. The electron exchange correlation functional was described by the Becke–Lee–Yang–Parr functional within the generalized gradient approximation framework. Relativistic effects were accounted for by employing an effective core potential, and atomic orbitals were represented using a double numerical polarization basis set [14]. To ensure effective convergence, a smearing value of 0.005 Ha was selected to accelerate the convergence process. The energy convergence criteria were set at 2 × 10^−5^ Ha, while the maximum force and maximum displacement convergence criteria were set at 0.004 Ha/Å and 0.005 Å, respectively. All calculations were performed at Center for Computational Chemistry and Molecular Simulation, College of Chemistry and Chemical Engineering, Southwest Petroleum University.

## 4. Conclusions

In this paper, based on the excellent properties of triphenylamine groups and hydrazone structure, a novel triphenylamine aldehyde salicylhydrazone molecule, DHBYMH, was successfully synthesized by using “triphenylamine” as the molecular framework and modifying it with a Suzuki coupling reaction and an aldoamine condensation reaction with salicylhydrazine. Its selective response to metal ions was investigated using UV and fluorescence spectra. The optical properties of the probe molecules were studied, and it was found that DHBYMH showed a highly polarity-distorted ICT mechanism based on the π-bridge induced by o-hydroxybenzene, which showed that the maximum fluorescence emission wavelength of the organic solvent showed an obvious redshift phenomenon, with the successive increases in the redshift degree with increasing polarity of the organic solvent. At the same time, the DHBYMH probe molecules also exhibited an ACQ effect, which is mainly due to the aggregation of probe molecules. As the aggregation degree increases, the molecular packing mode changes, resulting in a change in the electron transition of π-conjugated molecules, resulting in fluorescence quenching. DHBYMH achieved a detection limit of 1.62 × 10^−7^ M by demonstrating exceptional selectivity and sensitivity towards Cu^2+^ ions in an optimum sample solvent system (DMSO/H_2_O, (*v*/*v* = 7/3); pH = 7.0; cysteine (Cys) concentration: 1 × 10^−4^ M). The response mechanism was determined by nuclear magnetic resonance titration experiments, high-resolution mass spectrometry, and DFT calculations. Finally, a molecular logic gate was successfully designed based on the reversible recognition of Cu^2+^ ions by DHBYMH in the presence of EDTA.

## Figures and Tables

**Table 1:** Optical data from probe DHBYMH in different polar organic solvents (λ_abs_—absorption maximum and λ_em_—emission maximum).

DHBYMH	λ_abs_ (nm)	*λ*_em_ (nm)	Intensity	Stokes Shift (nm)	QY (%)
Toluene	380	454	461	74	15.64
Ethyl acetate	380	476	1603	96	18.51
THF	381	479	1994	98	19.27
Ethanol	386	501	690	115	22.31
DMF	390	514	1343	124	24.19
DMSO	384	520	3833	136	25.06

**Table 2:** Molecular logic gate truth table (Cu^2+^ and EDTA as chemical inputs, fluorescence intensity as chemical output).

Entry	Input A (Cu^2+^)	Input B (EDTA)	Output (Fluorescence)
1	0	0	1
2	0	1	1
3	1	0	0
4	1	1	1

## Data Availability

The data presented in this study are available in article and Supplementary Materials.

## Supplementary Materials

The following supporting information can be downloaded at: https://www.mdpi.com/article/10.3390/molecules29092032/s1, Figure S1: Ultraviolet absorption spectra (a) and fluorescence emission spectra (b) of DHBYMH (1 × 10^−5^ M) in different ratios of H_2_O/DMS. (c) Physical images of DHBYMH (1 × 10^−5^ M) in different ratios of H_2_O/DMSO under UV lamp (365 nm) irradiation. Figure S2: Lippert−Mataga plot for DHBYMH in various solvents. Figure S3: DHBYMH (1 × 10^−5^ M, DMSO/H_2_O, v/v = 7/3, pH = 7.0, Cys: 1 × 10^−4^ M) recognition of Cu^2+^ ions response time. Figure S4: FT-IR of DHB; Figure S5: ^1^H NMR (400 MHz, DMSO-d_6_) spectrum of DHB; Figure S6: ^13^C NMR (101 MHz, DMSO-d_6_) spectrum of DHB; Figure S7: FT-IR of DHBYMH; Figure S8: ^1^H NMR (400 MHz, DMSO-d_6_) spectrum of DHBYMH; Figure S9: ^13^C NMR (101 MHz, DMSO-d_6_) spectrum of DHBYMH; Figure S10: HR-MS spectra of DHBYMH. Table S1: The Stokes shift of DHBYMH in various solvents with a range of Δf (orientation polarizability) values. Table S2: Comparison of probe DHBYMH with other Cu^2+^ probes reported in the literature. Refs. [44,45,46] are cited in Supplementary Materials.
