# Supplementary material for: Synthesis and Application of Salicylhydrazone Probes with High Selectivity for Rapid Detection of Cu2+
Source: Molecules. 2024 Apr 28;29(9):2032. doi: 10.3390/molecules29092032 (PMC11085586; doi:10.3390/molecules29092032)
Supplement: Supplementary file 1 [file molecules-29-02032-s001.zip › molecules-2919503-supplementary.pdf]

Synthesis and Application of Salicylhydrazone Probes with High Selectivity for Rapid Detection of Cu^{2+}

Tianzhu Shi [1,2,*], Zhengfeng Xie [2], Xinliang Mo [1], Yulong Feng [1,*], Tao Peng [1], Fuyong Wu [1], Mei Yu [1], Jingjing Zhao [1], Li Zhang [1] and Ju Guo [1]

[1] Department of Brewing Engineering, Moutai Institute, Renhuai 564500, China; xinliangmo@163.com (X.M.); edifcztony@126.com (T.P.); wufuyong@mtxy.edu.cn (F.W.); yumei@mtxy.edu.cn (M.Y.); zhaojingjing0613@163.com (J.Z.); zhangli0026@126.com (L.Z.); guoju@mtxy.edu.cn (J.G.)

[2] Oil & Gas Field Applied Chemistry Key Laboratory of Sichuan Province, College of Chemistry and Chemical Engineering, Southwest Petroleum University, Chengdu 610500, China; xiezhf@swpu.edu.cn

* Correspondence: shitianzhu@mtxy.edu.com (T.S.); fengyulong@mtxy.edu.com (Y.F.);
Tel.: +86-185-8642-0308 (T.S.); +86-185-8573-8701 (Y.F.)

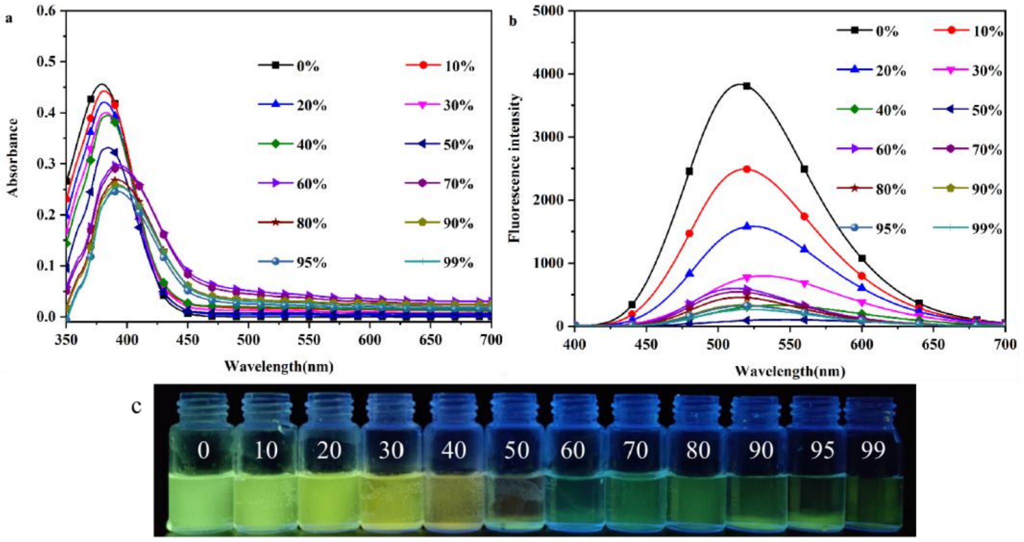

Figure S1 Ultraviolet absorption spectra (a) and Fluorescence emission spectra (b) of DHBYMH (1×10^{-5} M) in different ratios of H_2O/DMS；(c) Physical images of DHBYMH (1×10^{-5} M) in different ratios of H_2O/DMSO under UV lamp (365 nm) irradiation.

The Lippert-Mataga equation [1]

$$\Delta v = v_A - v_F = \frac{2\Delta\mu^2\Delta f}{hca_0^3} + \text{const} \tag{S1}$$

$$\Delta\mu = \mu_e - \mu_g \tag{S2}$$

$$\Delta f = \frac{\varepsilon-1}{2\varepsilon+1} - \frac{n^2-1}{2n^2+1} \tag{S3}$$

$$a_0 = \sqrt[3]{\frac{3M}{4N\pi d}} \tag{S4}$$

where Δν is the Stokes shift (in cm^{-1}) between the absorption and fluorescence emission peak, *h* is the Planck constant, *c* is the velocity of light in vacuum, $\Delta\mu$ is the transition dipole moments between the ground (μ_g) and excited (μ_e) states, Δf is the orientation polarizability of the solvents expressed by the dielectric constant (ε) and refractive index (n) of the solvents and a_0 is the Onsager cavity radius, which can be derived from the Avogadro number *N*, molecular weight *M*, and density *d* (*d* = 1.0 g/cm^3). The Onsager cavity radius (a_0) of DHBYMH was calculated to be 5.83 nm according to the equation (S4).

Obviously, the experimental data do not obey the linear relationship predicted by the Lippert-Mataga equation well, in the whole range of solvent polarity, as shown in Figure S2. A transformation in the slope of the ftted line is observed between the toluene (f=0.014)and ethanol (f= 0.295) solvents. Through the analysis of the fitted line in high-polarity solvents, a slope value of ≈4095.08 (R^2=0.77) was obtained.

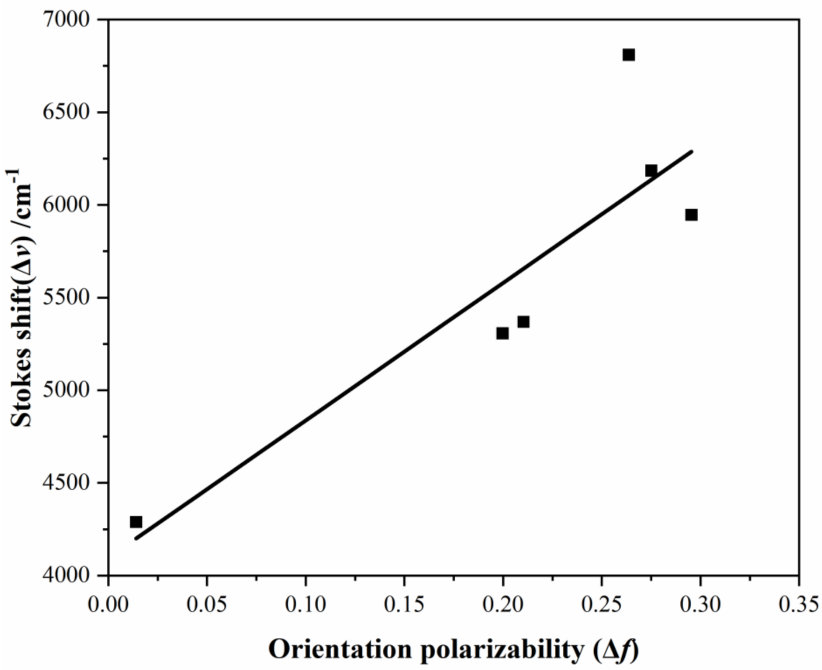

Figure S2 Lippert–Mataga plot for DHBYMH in various solvents.

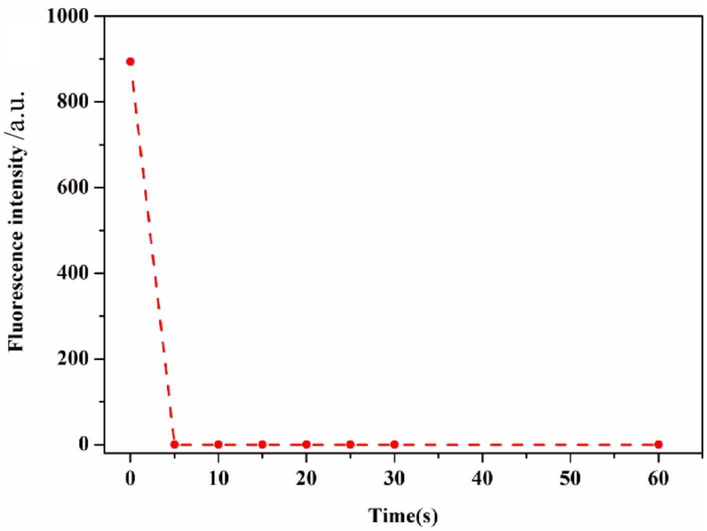

Figure S3 DHBYMH(1×10^{-5} M, DMSO/H_2O, v/v=7/3, pH=7.0, Cys: 1×10^{-4} M) recognition Cu^{2+} ion response time.

Optical study

The maximum UV absorption wavelength of DHBYMH in in different polar organic solvents (toluene, ethyl acetate, THF, ethanol, DMF, and DMSO) was used as the excitation wavelength for measuring the fluorescence wavelength. UV–vis spectra were recorded using UV-2450 type ultraviolet-visible spectrophotometer. Photoluminescence (PL) spectra were obtained using RF-5301PC fluorescence spectrophotometer. The

photoluminescence quantum yields (QY) in solutions were determined by comparing to quinine sulfate in 0.10 M H_2SO_4 at room temperature as a standard. The QY in solid states were obtained on a HORIBA Fluorolog-3 spectrophotometer equipped with an integrating sphere.

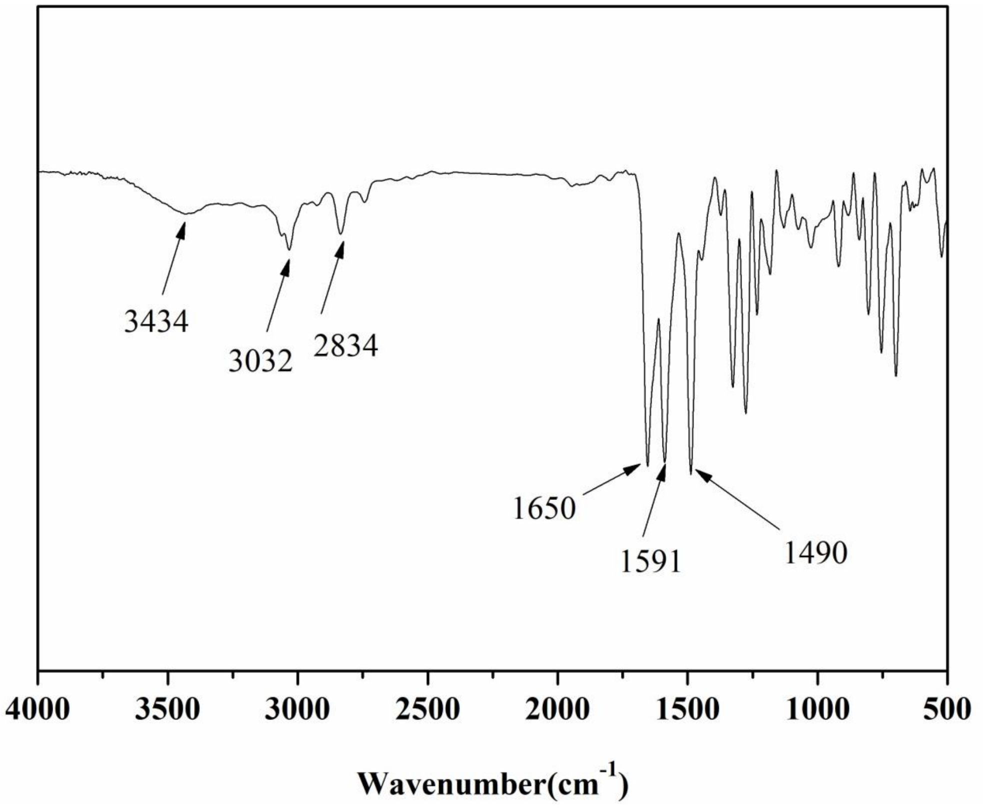

Figure S4 FT-IR of DHB

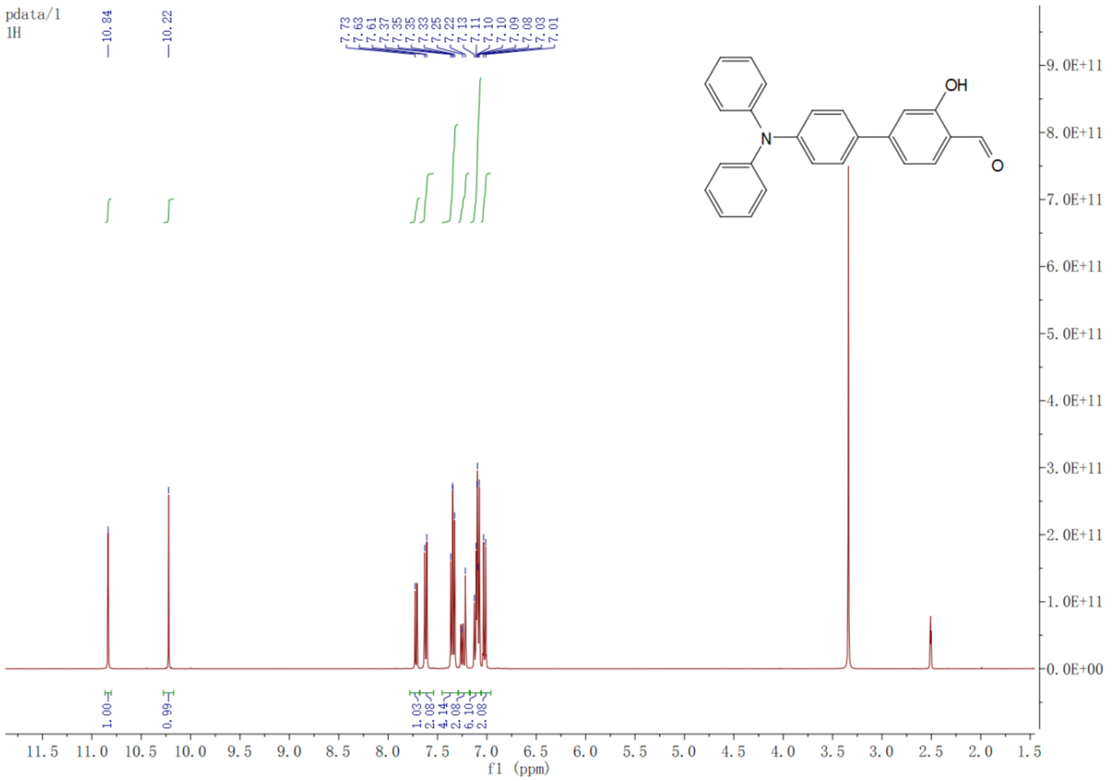

Figure S5 ^{1}H NMR (400 MHz, DMSO-d_6) spectrum of DHB

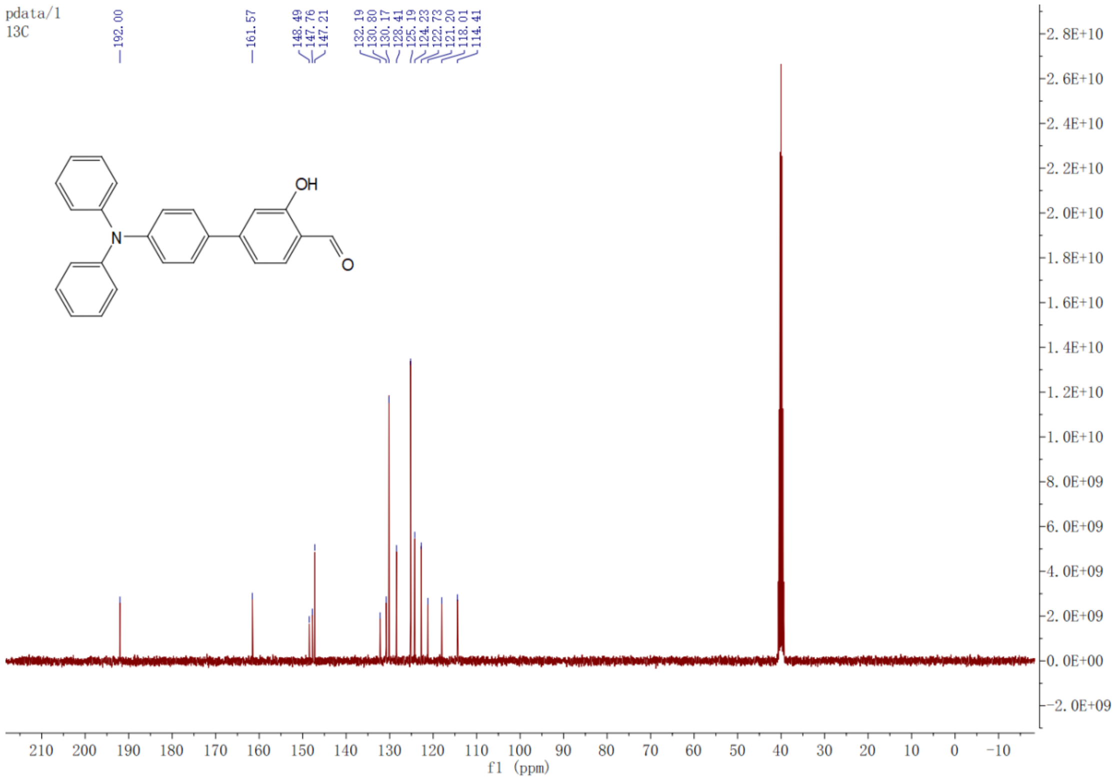

Figure S6 ^{13}C NMR (101 MHz, DMSO-d_6) spectrum of DHB

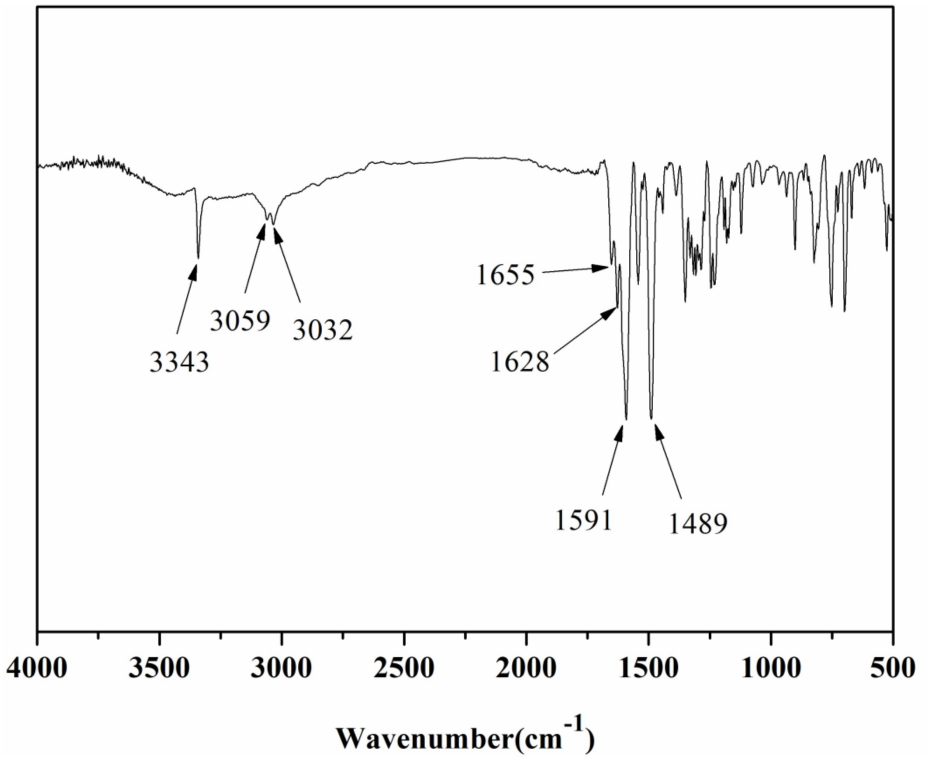

Figure S7FT-IR of DHBYMH

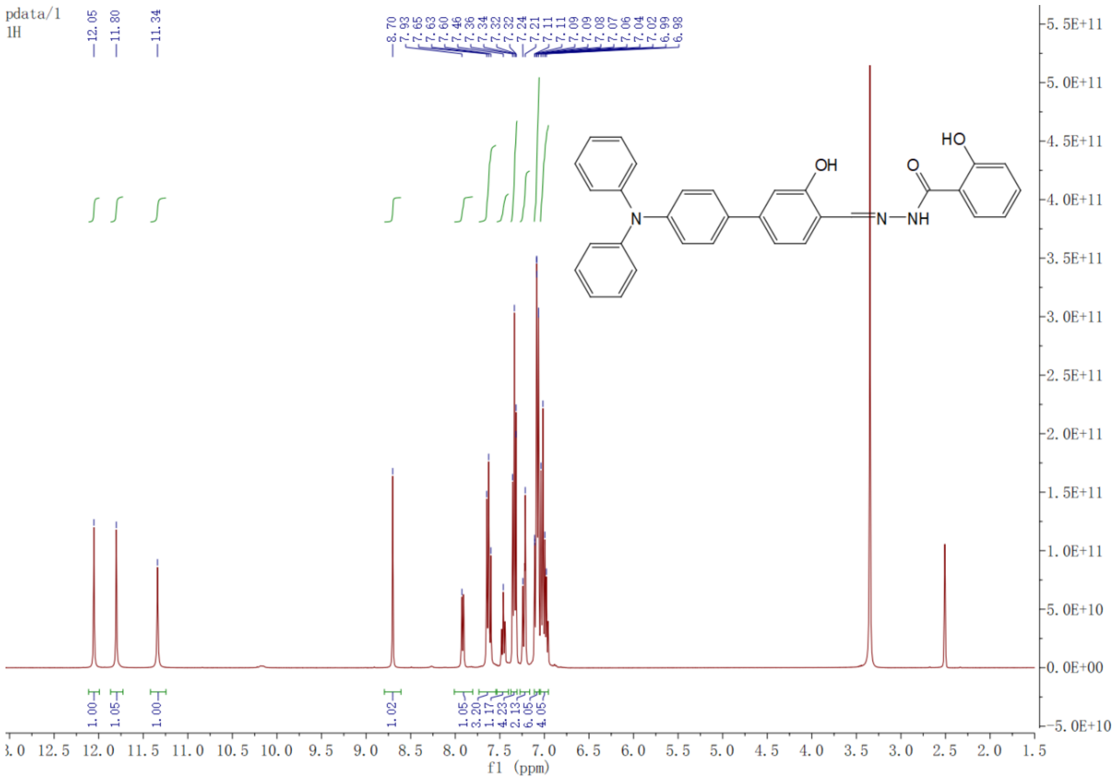

Figure S8 ^{1}H NMR (400 MHz, DMSO-d_6) spectrum of DHBYMH

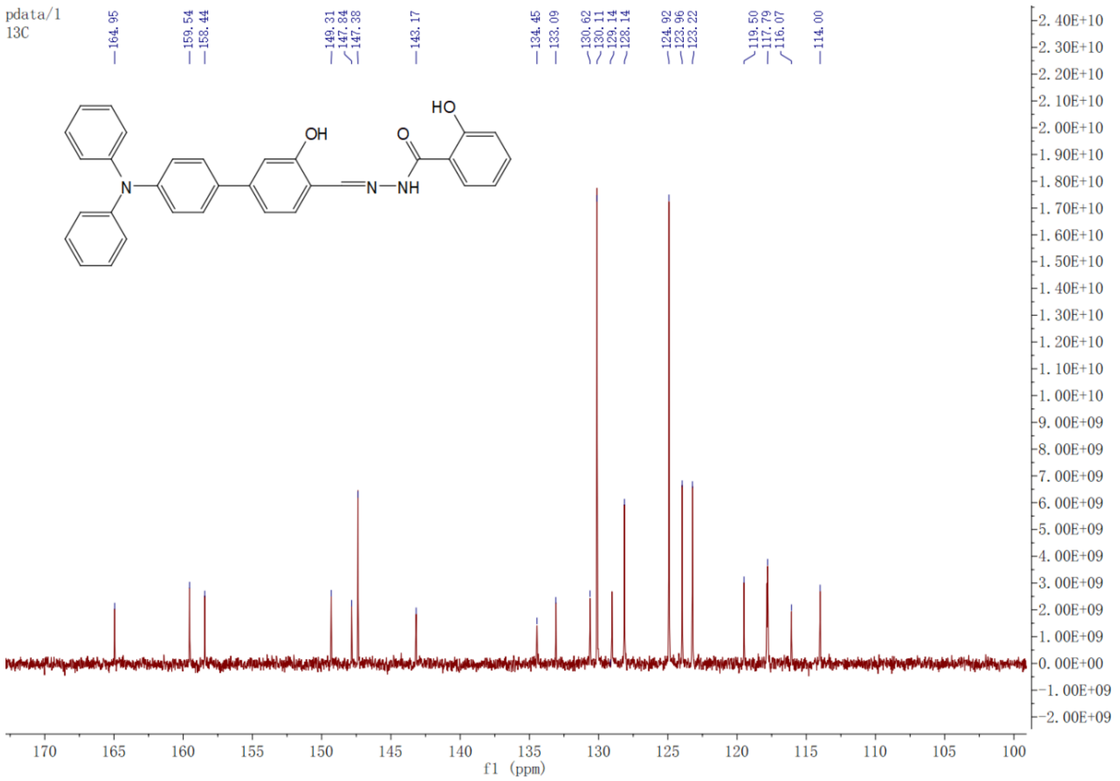

Figure S9 ^{13}C NMR (101 MHz, DMSO-d_6) spectrum of DHBYMH

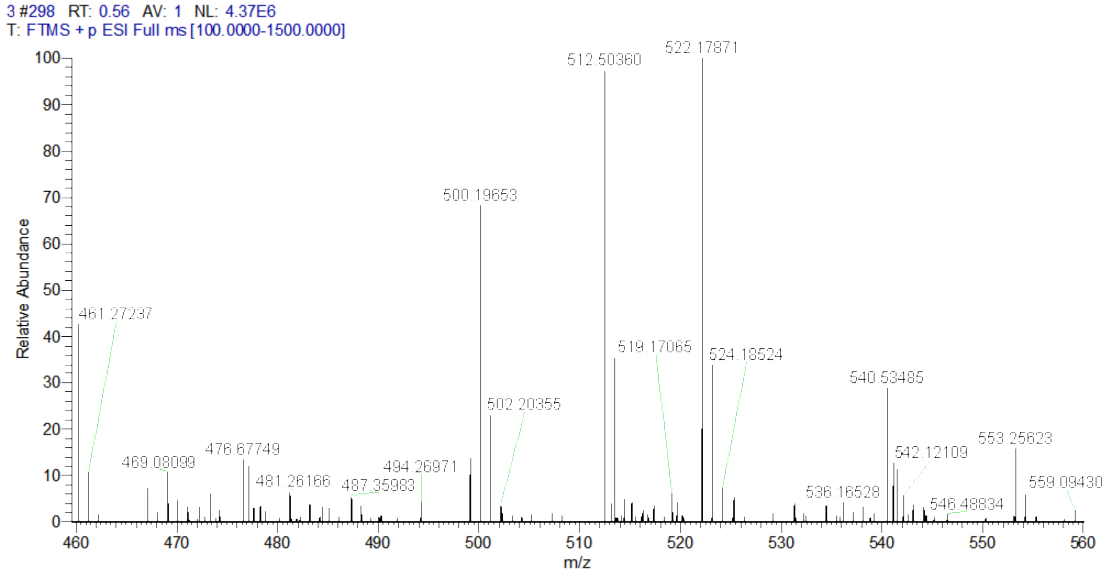

Figure S10 HR-MS spectra of DHBYMH

Table S1. The Stokes shift of DHBYMH in various solvents with a range of Δf (orientation polarizability) values.

DHBYMH	λ_{abs}(nm)	λ_{em}(nm)	n	Stokes shift(cm^{-1})	ε	f
Toluene	380	454	1.494	4289	2.38	0.014
Ethyl acetate	380	476	1.372	5307	6.02	0.199
THF	381	479	1.405	5369	7.58	0.210
Ethanol	386	501	1.354	5946	28.4	0.295
DMF	390	514	1.42817	6185	36.7	0.275
DMSO	384	520	1.4795	6810	48.9	0.264

Table S2 Comparison of probe DHBYMH with other Cu^{2+} probes reported.

Probe	Solvent (V:V)	LOD/(mol• L^{-1})	pH	Time	Cell	Ref.
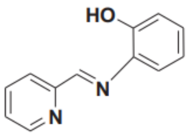	H_2O/methanol (9:1 v/v)	2.00×10^{-5}	7	60 s	—	[2]
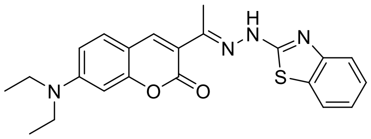	CH_3CN/H_2O (1:1)	5.80×10^{-8}	7.2	1 h	MCF-7 cells	[3]
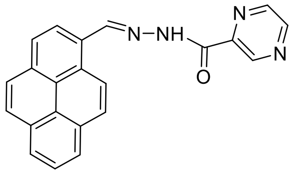	ACN/PBS (4:6)	1.57×10^{-7}	7.4	—	Hela cells	[4]
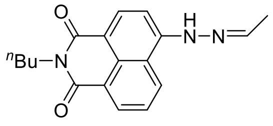	CH_3CN/HEPES (4:1)	3.20×10^{-7}	7.4	20 min	293 T cells	[5]
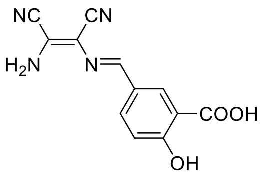	CH_3CN/HEPES (1:9)	2.19×10^{-7}	7.0	11 min	HepG2 cells	[6]

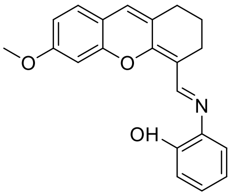	CH_3CN/HEPES (4:1)	3.30×10^{-5}	7.3	—	HepG2 cells	[7]
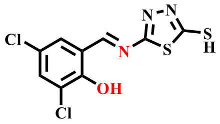 SB-1	H_2O	4.48×10^{-6}	4.2-10.5	150 s	Caco-2 cells	[8]
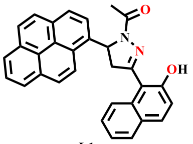 L1	EtOH–H_2O =1:1	1.62×10^{-6}	—	—	—	[9]
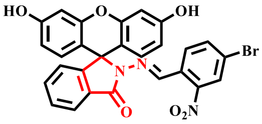 A5	PBS buffer/ EtOH (1:1)	0.11 μM	7.4	120s	—	[10]
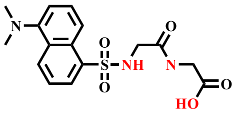 GG-Dns	CH_3CN/H_2O (1/1)	2.9×10^{-7}	1.0-3.5	—	—	[11]
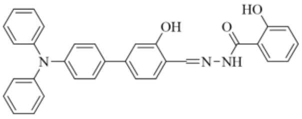	DMSO/H_2O (7/3）	1.62×10^{-7} M	7.0	5s		**This study**

1. Cao, J.; Liu, Q.-M.; Bai, S.-J.; Wang, H.-C.; Ren, X.; Xu, Y.-X. Ladder-Type Dye with Large Transition Dipole Moment for Solvatochromism and Microphase Visualization. *ACS Applied Materials & Interfaces* **2019**, *11*, 29814-29820, doi:10.1021/acsami.9b07677.
2. Lina, G.; Gao, Y.; Han, L. Detecting Cu2+ and H2O in methanol based on aggregation-induced emission fluorescent enhancement. *Journal of Coordination Chemistry* **2021**, *74*, 1284-1297, doi:10.1080/00958972.2021.1897114.
3. Zhang, Z.; Liu, Y.; Wang, E. A highly selective “turn-on” fluorescent probe for detecting Cu2+ in two different sensing mechanisms. *Dyes and Pigments* **2019**, *163*, 533-537, doi:10.1016/j.dyepig.2018.12.039.
4. Yin, J.; Wang, Z.; Zhao, F.; Yang, H.; Li, M.; Yang, Y. A novel dual functional pyrene-based turn-on fluorescent probe for hypochlorite and copper (II) ion detection and bioimaging applications. *Spectrochimica Acta Part A: Molecular and Biomolecular Spectroscopy* **2020**, *239*, doi:10.1016/j.saa.2020.118470.
5. Fu, Y.; Pang, X.-X.; Wang, Z.-Q.; Chai, Q.; Ye, F. A highly sensitive and selective fluorescent probe for determination of Cu (II) and application in live cell imaging. *Spectrochimica Acta Part A: Molecular and Biomolecular Spectroscopy* **2019**, *208*, 198-205, doi:10.1016/j.saa.2018.10.005.

6. Jiang, N.; Gong, X.; Zhong, T.; Zheng, Y.; Wang, G. A highly selective and sensitive “turn-on” fluorescent probe for rapid recognition and detection of Cu2+ in aqueous solution and in living cells. *Journal of Molecular Structure* **2020**, *1219*, doi:10.1016/j.molstruc.2020.128573.
7. Li, B.; Kou, J.; Mei, H.; Gu, X.; Wang, M.; Xie, X.; Xu, K. A hemicyanine-based “turn-on” fluorescent probe for the selective detection of Cu2+ ions and imaging in living cells. *Analytical Methods* **2020**, *12*, 4181-4184, doi:10.1039/d0ay01461c.
8. Zhang, C.; Wang, Y.; Li, X.; Nie, S.; Liu, C.; Zhang, Y.; Guo, J.; Liu, C. A dual functional fluorescent probe based on naphthalimide for detecting Cu2+ and pH and its applications. *Inorganica Chimica Acta* **2023**, *554*, doi:10.1016/j.ica.2023.121544.
9. Yang, Y.-S.; Cao, J.-Q.; Ma, C.-M.; Zhang, Y.-P.; Guo, H.-C.; Xue, J.-J. A novel pyrazoline-based fluorescence probe armed by pyrene and naphthol system for the selective detection of Cu2+ and its biological application. *Journal of the Iranian Chemical Society* **2022**, *19*, 3451-3461, doi:10.1007/s13738-022-02536-5.
10. Leng, X.; Wang, D.; Mi, Z.; Zhang, Y.; Yang, B.; Chen, F. Novel Fluorescence Probe toward Cu2+ Based on Fluorescein Derivatives and Its Bioimaging in Cells. *Biosensors* **2022**, *12*, doi:10.3390/bios12090732.
11. Wang, Y.; Zhou, J.; Zhao, L.; Xu, B. A dual-responsive and highly sensitive fluorescent probe for Cu2+ and pH based on a dansyl derivative. *Dyes and Pigments* **2020**, *180*, doi:10.1016/j.dyepig.2020.108513.
